# RNA-based stable isotope probing provides no indication for rapid α-synuclein assimilation by murine gut bacteria

**DOI:** 10.1099/acmi.0.000345

**Published:** 2022-05-04

**Authors:** Lena Brandau, Severin Weis, Maximilian Weyland, Fabian K. Berger, Sylvia Schnell, Karl-Herbert Schäfer, Markus Egert

**Affiliations:** ^1^​ Faculty of Medical and Life Sciences, Institute of Precision Medicine, Microbiology and Hygiene Group, Furtwangen University, Jakob-Kienzle-Straße 17, Villingen-Schwenningen, Germany; ^2^​ Department of Informatics and Microsystem Technology, University of Applied Sciences Kaiserslautern, Working Group Enteric Nervous System, Amerikastraße 1, Zweibrücken, Germany; ^3^​ Institute for Medical Microbiology and Hygiene, Saarland University, Kirrberger Straße 100, Homburg/Saar, Germany; ^4^​ German National Reference Centre for *Clostridioides (Clostridium) difficile* , Homburg/Saar-Münster-Coesfeld, Germany; ^5^​ Research Centre for BioSystems, Land Use, and Nutrition (IFZ), Institute of Applied Microbiology, Justus-Liebig-University Giessen, Giessen, Germany

**Keywords:** Parkinson’s disease, protein degradation, intestinal bacteria

## Abstract

In Parkinson’s disease (PD), α-synuclein is a key protein in the process of neurodegeneration. Besides motor symptoms, most PD patients additionally suffer from gastrointestinal tract (GIT) dysfunctions, even several years before the onset of motor disabilities. Studies have reported a dysbiosis of gut bacteria in PD patients compared to healthy controls and have suggested that the enteric nervous system (ENS) can be involved in the development of the disease. As α-synuclein was found to be secreted by neurons of the ENS, we used RNA-based stable isotope probing (RNA-SIP) to identify gut bacteria that might be able to assimilate this protein. The gut contents of 24 mice were pooled and incubated with isotopically labelled (^13^C) and unlabelled (^12^C) α-synuclein. After incubation for 0, 4 and 24 h, RNA was extracted from the incubations and separated by density gradient centrifugation. However, RNA quantification of density-resolved fractions revealed no incorporation of the ^13^C isotope into the extracted RNA, suggesting that α-synuclein was not assimilated by the murine gut bacteria. Potential reasons and consequences for follow-up-studies are discussed.

## Data Summary

The authors confirm supporting data, code and protocols have been provided within the article or through supplementary data files or are available from the corresponding author on reasonable request.

## Introduction

Parkinson’s disease (PD) is one of the most common neurodegenerative diseases and even today there is still no treatment available that can completely cure affected patients [1]. PD patients not only suffer from motor symptoms, but in most cases also from gastrointestinal dysfunctions [2] that can arise up to 20 years before the onset of the typical motor symptoms [3, 4]. An early diagnosis of these non-motor symptoms, with immediate treatment, could therefore make a significant contribution to protecting neuronal function from degeneration, slowing the course of the disease and enabling patients to improve their quality of life [5].

The α-synuclein protein plays an essential role in the development of PD, although the detailed mechanisms are not yet fully understood [6]. As a typical histological characteristic in most PD patients, α-synuclein is present in an altered structure or is overexpressed, leading to aggregation of the molecules, which promotes the formation of so-called Lewy bodies and neurodegenerative processes [7]. In addition to other α-synucleopathies, PD in particular has been reported to come with increased expression of α-synuclein in the enteric nervous system [8–10], suggesting that the synucleinopathy arises first in the gastrointestinal tract (GIT) and then spreads to the central nervous system of the brain at a later stage of the disease [11]. A recent study in a PD mouse model demonstrated alterations in both ENS composition and gut motility at very early timepoints, while the brain did not yet show significant changes [12]. Paillusson *et al*. reported that enteric neurons are physiologically secreting α-synuclein [13], which could potentially make it reachable for gut bacteria and serve as a seed for further propagation. The microbiota–gut–brain axis seems to play an important role in PD, as researchers have found microbial dysbiosis in stool samples of PD patients [14–19]. However, it is not clear yet whether such dysbiosis is rather a cause or a consequence of the disease [5].

In this study, we aimed to use RNA-based stable isotope probing (RNA-SIP) to identify potentially synuclein-assimilating bacteria in the gut contents of mice being used as model organisms. RNA-SIP is an elegant technique to unravel which groups of microorganisms assimilate a certain substrate within a complex community [20–22]. We previously used this technique to successfully identify prebiotics-degrading intestinal bacteria [23]. We believe that the potential identification of (specifically) synuclein-degrading bacteria from the intestinal contents of mice and (later on) humans might contribute to a deeper understanding of the interactions between PD and the intestinal ecosystem and might even facilitate an earlier diagnosis of this complex disease. A great advantage of RNA-SIP is that no radioactive labels are needed [22, 23]. Instead, microbial samples are incubated with substrates containing stable isotopes. Microorganisms that are able to assimilate these substrates utilize the isotopes during their metabolism and biosynthesis and incorporate the label into their RNA [22]. After extraction, labelled and unlabelled RNA can be separated by isopycnic density gradient ultracentrifugation and the resulting gradients are collected in fractions of descending density. After recovery of the RNA, standard high-throughput technologies can be used to unravel differences in community compositions between the labelled an unlabelled fractions and identify specific bacteria that have assimilated the provided substrate [23].

## Methods

### Collection and incubation of intestinal content, and RNA extraction

Twenty-five adult Balb/c mice of both sexes were reared in the animal house of experimental surgery at the Saarland University Medical Centre (Homburg/Saar, Germany) under standard laboratory conditions with a 12 h light/dark rhythm and were fed on a standard maintenance diet for rats and mice (1324–10 mm pellets; Altromin Spezialfutter GmbH and Co. KG, Lage, Germany). Both food and drinking water were available to the mice *ad libitum*. Animals were killed by cervical dislocation. The large intestines of the animals were dissected and brought to an anaerobic work bench. From that point, the intestines and contents were handled under anaerobic conditions.

Murine caecal contents were collected and pooled in M9 minimal medium [24] as a 15 % (w/v) slurry under anaerobic conditions, as described previously, but without glucose as carbon source [25]. For each incubation sample, 1 ml of faecal slurry was mixed with 1 ml M9 minimal medium containing 100 µM of either unlabelled α-synuclein (AlexoTech, Umea, Sweden) or uniformly ^13^C-labelled α-synuclein (AlexoTech). All samples were incubated at 37 °C under anaerobic conditions for 0, 4 or 24 h in triplicate, respectively, and then immediately frozen and stored at −80 °C.

Total RNA from each sample was extracted using the RNeasy Power Microbiome kit containing an on-column DNase I treatment (Qiagen, Hilden, Germany) following the manufacturer’s instructions. Follow-up in-solution DNase treatment was performed with the Monarch Total RNA Miniprep kit (New England Biolabs, Frankfurt am Main, Germany) according to the manufacturer’s instructions. The three replicate incubations per time point were finally combined during the DNase treatment and absence of DNA was verified by PCR of the 16S rRNA gene using 0.25 µl of each conventional 27F and 519R primer (50 µM) [26, 27], 0.5 µl of dNTP mix (10 mM), 2.5 µl of 10× DreamTaq buffer (containing 20 mM MgCl_2_), 0.125 µl DreamTaq DNA polymerase (5 U µl^−1^), 20.375 µl nuclease-free water and 1 µl of template. The thermal profile was set as described previously [28], using 34 instead of 25 cycles.

### Density gradient centrifugation, gradient fractionation and fraction analysis

For density gradient centrifugation, centrifugation tubes (Polypropylene Bell-Top Quick-Seal Centrifuge Tubes, Beckman Coulter, Krefeld, Germany) were filled with 8.5 ml of a centrifugation mix containing 6.739 ml cesium trifluoroacetate (CsTFA, 2.0 ± 0.05 g ml^−1^, GE Healthcare, Freiburg, Germany), 1.498 ml gradient buffer [29] and 0.263 ml nuclease-free formamide (Carl Roth GmbH, Karlsruhe, Germany). The refractive index of the mix was set to 1.3724 with an AR200 digital refractometer (Reichert, Depew, NY, USA) and RNA was added to 100 ng ml^−1^ [22]. Centrifugation was performed at 123, 100 ***g*** and 20 °C for 68 h using the Optima MAX-XP Ultracentrifuge with an MLN-80 near vertical Rotor (Beckman Coulter).

After centrifugation, each gradient sample was collected in 16 fractions of 0.5 ml by replacement with water [28]. From a 75 µl aliquot of each fraction the density and refractive index were determined. These parameters were correlated to generate a calibration curve.

### RNA recovery, quantification, reverse transcription and cDNA analysis

RNA was precipitated from the CsTFA mix by isopropanol–ethanol precipitation [22]. RNA quantification of the fractions was conducted with a microplate reader (Infinite 200 PRO, Tecan, Männedorf, Switzerland) using the Quant-it RiboGreen RNA Assay kit (Thermo Fisher Scientific, Waltham, MA, USA) according to the manufacturer’s instructions. The RNA concentration was correlated with the fraction number and the density, which had been calculated previously by means of a calibration curve.

Reverse transcription of the RNA was performed with the SuperScript VILO cDNA Synthesis kit (Thermo Fisher Scientific), according to manufacturer’s instructions. The cDNA was amplified in accordance with the same protocol as mentioned above, using 25 PCR-cycles.

PCR products were analysed by standard gel electrophoresis, using a 0.8 % agarose gel and Midori Green Advance (Biozym, Hessisch Oldendorf, Germany) as DNA dye.

## Results and discussion

A synopsis of recent studies suggests that there are small but robust differences in the bacterial community composition of PD patients in comparison to healthy controls [30]. While genera such as *Lactobacillus *, *Akkermansia* and *Bifidobacterium* were relatively enriched, bacteria belonging to the family *Lachnospiraceae* and the genus *Faecalibacterium *, both short-chain fatty acid producers, were relatively depleted. Such dysbiosis might lead to a proinflammatory status, which could be linked to the recurrent gastrointestinal symptoms affecting PD patients. In addition, differences in microbiota composition might not only be of therapeutic but also of diagnostic value, preparing a new basis for an early diagnosis of PD using faecal microbiota analyses. We hypothesized that specifically α-synuclein-degrading bacteria might be of particular diagnostic interest and set up a pilot RNA-SIP study using murine faecal samples and fully ^13^C-labelled α-synuclein as substrate.

**Fig. 1.**
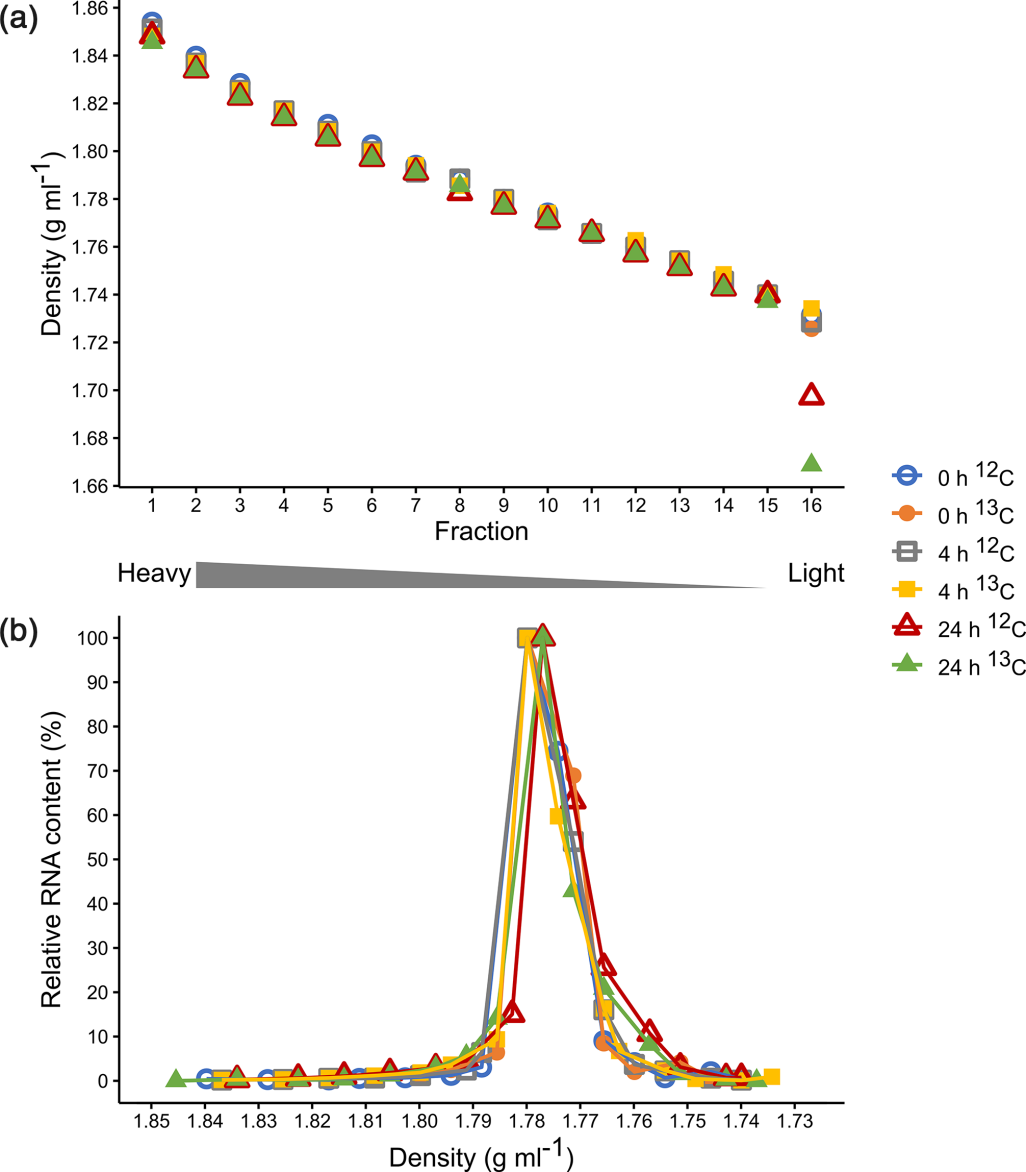
Density gradients and RNA contents. The formation of the density gradients (**a**) resulted in a linear distribution with an averaged range from 1.8492 (fraction 1) to 1.7143 g ml^−1^ (fraction 16). (**b**) Density-dependent distribution of RNA. RNA was extracted from murine gut contents, incubated with ^12^C α-synuclein and ^13^C α-synuclein for 0, 4 and 24 h, respectively, and separated by ultracentrifugation using a CsTFA density gradient. RNA concentration is given as relative RNA content, whereby the highest RNA concentration of each gradient is set as 100 % (543.50 ng ml^−1^ 0 h ^12^C, 487.98 ng ml^−1^ 0 h ^13^C, 497.92 ng ml^−1^ 4 ^12^C, 424.94 ng ml^−1^ 4 h ^13^C, 326.45 ng ml^−1^ 24 h ^12^C, 365.21 ng ml^−1^ 24 h ^13^C).

After ultracentrifugation, the obtained density gradients for the six incubation samples ranged from 1.8492 (fraction 1) to 1.7143 g ml^−1^ (fraction 16) (Fig. 1a). The density distribution of the gradient fractions showed a linear trend. This linear character among the gradients as well as the range of the determined densities was very similar to previous reports [28, 29, 31–33]. Clearly, the obtained CsTFA gradients were appropriate for the separation of stable isotope-labelled RNA from unlabelled RNA. The density of the 24 h incubations dropped at the last ‘light’ fraction (fraction 16). This was most likely caused by dilution of the fraction with water, as fraction 16 is the last fraction of the gradient on the boundary to the water that is used for fractionation [23].

After RNA extraction from the gradient fractions, the quantification showed one peak in the RNA distribution over the gradient fractions (Fig. 1b). The highest RNA concentrations were found in fraction 9 at an average density of 1.7789 g ml^−1^ for all incubation samples. From previous publications it is known that unlabelled RNA usually peaks around this density [22, 25, 28, 32]. In case of any ^13^C assimilation into RNA, we would have expected to see shifts of the distribution curves towards higher densities, particularly for the 4 and 24 h incubations with ^13^C synuclein. However, there was no difference in RNA distribution between both control incubations (0 h) and ^12^C and the ^13^C incubations. In fact, all distribution curves were nearly identical.

**Fig. 2.**
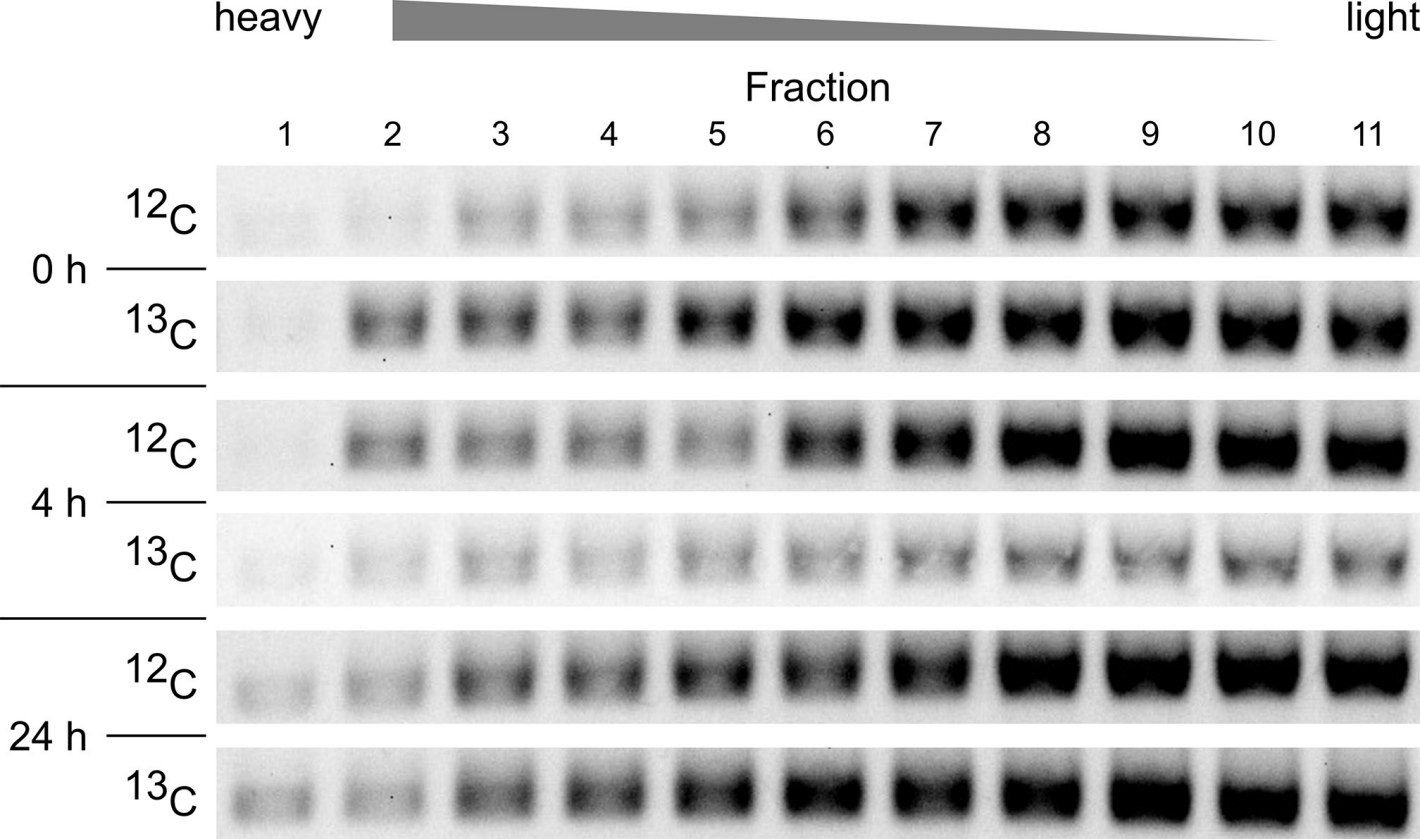
Banding patterns of 16S rRNA gene amplicons, derived from cDNA of fractions 1–11 collected from the six RNA-SIP gradients (for details see text or legend of Fig. 1). The figure shows a combination of six single agarose gels. All bands are ∼500 bp.

Gel electrophoresis of 16S rRNA gene amplicons, produced with cDNA from the gradient fractions 1–12 as template, showed the same pattern (Fig. 2). Despite some variations, the thickness of the bands (all representing amplicons of ~500 bp) did not suggest any clear increase of amplicon concentration in the heavy fractions of the ^13^C incubations in comparison to the respective ^12^C incubations. For all inspected gradients, the largest amount of cDNA was obviously present in fractions 8 to 11, corroborating the RNA distribution determined by means of RiboGreen (Fig. 1).

Our failure to determine any formation of isotopically labelled RNA suggests that α-synuclein had not been incorporated into the RNA of the murine gut bacteria. As to the best of our knowledge, RNA-SIP has never been used to analyse the degradation and assimilation of ^13^C-labelled proteins before, we can only speculate on potential reasons. The used concentration of α-synuclein might have been too low and/or the incubation time too short to allow for sufficient protein degradation and assimilation into the bacterial RNA. In addition, competition of α-synuclein with residual (unlabelled) substrates stemming from the intestinal tract samples might also have hampered the production of isotopically labelled RNA. Moreover, incorporation just might have been too low to be measurable with the methods applied here. However, in previous RNA-SIP studies with complex carbohydrates (resistant potato starch), isotopically labelled RNA from murine intestinal bacteria was detectable within 2–4 h of incubation, albeit using relatively high substrate concentrations (40 mM glucose equivalents) [25]. Clearly, more basic studies dealing with the degradation and potential assimilation of synuclein into bacterial RNA are needed to prepare follow-up SIP studies, maybe also involving pure cultures instead of complex intestinal communities as well as other labelled proteins as control substrates. Recently, PLFA-(poly unsaturated lipid fatty acid) and AA-(amino acid)SIP were successfully used to identify Gram-positive bacteria as controllers of protein degradation in soils [34], suggesting that other SIP techniques, including protein-SIP [35], may be more suitable to follow protein degradation and assimilation than RNA-SIP.

Finally, it might also be speculated that human faecal bacteria, in particular those from PD patients, might be more adapted to α-synuclein degradation than bacteria from mice, which usually thrive on a carbohydrate-rich diet. Therefore, SIP follow-up experiments using human (faecal) bacteria appear particularly reasonable to elucidate whether α-synuclein is specifically metabolized by human intestinal microorganisms.

## Conclusion

Using RNA-SIP, no assimilation of α-synuclein (50 µM) by murine gut bacteria within 24 h could be shown. We nevertheless believe that the identification of specifically α-synuclein-assimilating intestinal bacteria might help to improve an early diagnosis of PD. Follow-up studies should consider our results regarding substrate concentration, incubation time, microbial inoculum and type of SIP technology.

## References

[1] Perez-Pardo P, Kliest T, Dodiya HB, Broersen LM, Garssen J (2017). The gut-brain axis in Parkinson’s disease: Possibilities for food-based therapies. Eur J Pharmacol.

[2] Pfeiffer RF (2018). Gastrointestinal dysfunction in Parkinson’s Disease. Curr Treat Options Neurol.

[3] Lang AE (2011). A critical appraisal of the premotor symptoms of Parkinson’s disease: potential usefulness in early diagnosis and design of neuroprotective trials. Mov Disord.

[4] Savica R, Carlin JM, Grossardt BR, Bower JH, Ahlskog JE (2009). Medical records documentation of constipation preceding Parkinson disease: A case-control study. Neurology.

[5] Nair AT, Ramachandran V, Joghee NM, Antony S, Ramalingam G (2018). Gut microbiota dysfunction as reliable non-invasive early diagnostic biomarkers in the pathophysiology of Parkinson’s disease: a critical review. J Neurogastroenterol Motil.

[6] Rocha EM, De Miranda B, Sanders LH (2018). Alpha-synuclein: Pathology, mitochondrial dysfunction and neuroinflammation in Parkinson’s disease. Neurobiol Dis.

[7] Houser MC, Tansey MG (2017). The gut-brain axis: is intestinal inflammation a silent driver of Parkinson’s disease pathogenesis?. NPJ Parkinsons Dis.

[8] Gold A, Turkalp ZT, Munoz DG (2013). Enteric alpha-synuclein expression is increased in Parkinson’s disease but not Alzheimer’s disease. Mov Disord.

[9] Shannon KM, Keshavarzian A, Dodiya HB, Jakate S, Kordower JH (2012). Is alpha-synuclein in the colon a biomarker for premotor Parkinson’s disease? Evidence from 3 cases. Mov Disord.

[10] Hilton D, Stephens M, Kirk L, Edwards P, Potter R (2014). Accumulation of α-synuclein in the bowel of patients in the pre-clinical phase of Parkinson’s disease. Acta Neuropathol.

[11] Braak H, de Vos RAI, Bohl J, Del Tredici K (2006). Gastric alpha-synuclein immunoreactive inclusions in Meissner’s and Auerbach’s plexuses in cases staged for Parkinson’s disease-related brain pathology. Neurosci Lett.

[12] Gries M, Christmann A, Schulte S, Weyland M, Rommel S (2021). Parkinson mice show functional and molecular changes in the gut long before motoric disease onset. Mol Neurodegener.

[13] Paillusson S, Clairembault T, Biraud M, Neunlist M, Derkinderen P (2013). Activity-dependent secretion of alpha-synuclein by enteric neurons. J Neurochem.

[14] Weis S, Schwiertz A, Unger MM, Becker A, Faßbender K (2019). Effect of Parkinson’s disease and related medications on the composition of the fecal bacterial microbiota. NPJ Parkinsons Dis.

[15] Unger MM, Spiegel J, Dillmann K-U, Grundmann D, Philippeit H (2016). Short chain fatty acids and gut microbiota differ between patients with Parkinson’s disease and age-matched controls. Parkinsonism Relat Disord.

[16] Hill-Burns EM, Debelius JW, Morton JT, Wissemann WT, Lewis MR (2017). Parkinson’s disease and Parkinson’s disease medications have distinct signatures of the gut microbiome. Mov Disord.

[17] Scheperjans F, Aho V, Pereira PAB, Koskinen K, Paulin L (2015). Gut microbiota are related to Parkinson’s disease and clinical phenotype. Mov Disord.

[18] Li W, Wu X, Hu X, Wang T, Liang S (2017). Structural changes of gut microbiota in Parkinson’s disease and its correlation with clinical features. Sci China Life Sci.

[19] Hopfner F, Künstner A, Müller SH, Künzel S, Zeuner KE (2017). Gut microbiota in Parkinson disease in a northern German cohort. Brain Res.

[20] Berry D, Loy A (2018). Stable-isotope probing of human and animal microbiome function. Trends Microbiol.

[21] Lueders T, Dumont MG, Bradford L, Manefield M (2016). RNA-stable isotope probing: from carbon flow within key microbiota to targeted transcriptomes. Curr Opin Biotechnol.

[22] Weis S, Schnell S, Egert M (2019). RNA-based stable isotope probing (RNA-SIP) in the gut environment. Methods Mol Biol.

[23] Egert M, Weis S, Schnell S (2018). RNA-based stable isotope probing (RNA-SIP) to unravel intestinal host-microbe interactions. Methods.

[24] Smith HO, Levine M (1964). Two sequential repressions of DNA synthesis in the establishment of lysogeny by phage P22 and its mutants. Proc Natl Acad Sci U S A.

[25] Herrmann E, Young W, Rosendale D, Conrad R, Riedel CU (2017). Determination of resistant starch assimilating bacteria in fecal samples of mice by in vitro RNA-based stable isotope probing. Front Microbiol.

[26] Lane DJ, Stackebrandt E, Goodfellow M (1991). Molecular Microbiological Methods: Nucleic Acid Techniques in Bacterial Systematics.

[27] Lane DJ, Pace B, Olsen GJ, Stahl DA, Sogin ML (1985). Rapid determination of 16S ribosomal RNA sequences for phylogenetic analyses. Proc Natl Acad Sci U S A.

[28] Herrmann E, Young W, Rosendale D, Reichert-Grimm V, Riedel CU (2017). RNA-based stable isotope probing suggests *Allobaculum* spp. as particularly active glucose assimilators in a complex murine microbiota cultured *in vitro*. Biomed Res Int.

[29] Young W, Egert M, Bassett SA, Bibiloni R (2015). Detection of sialic acid-utilising bacteria in a caecal community batch culture using RNA-based stable isotope probing. Nutrients.

[30] Romano S, Savva GM, Bedarf JR, Charles IG, Hildebrand F (2021). Meta-analysis of the Parkinson’s disease gut microbiome suggests alterations linked to intestinal inflammation. NPJ Parkinsons Dis.

[31] Egert M, de Graaf AA, Maathuis A, de Waard P, Plugge CM (2007). Identification of glucose-fermenting bacteria present in an in vitro model of the human intestine by RNA-stable isotope probing. FEMS Microbiol Ecol.

[32] Whiteley AS, Thomson B, Lueders T, Manefield M (2007). RNA stable-isotope probing. Nat Protoc.

[33] Herrmann E, Young W, Reichert-Grimm V, Weis S, Riedel CU (2018). *In vivo* assessment of resistant starch degradation by the caecal microbiota of mice using RNA-based stable isotope probing-a proof-of-principle study. Nutrients.

[34] Enggrob KL, Larsen T, Peixoto L, Rasmussen J (2020). Gram-positive bacteria control the rapid anabolism of protein-sized soil organic nitrogen compounds questioning the present paradigm. Sci Rep.

[35] Jehmlich N, Vogt C, Lünsmann V, Richnow HH, von Bergen M (2016). Protein-SIP in environmental studies. Curr Opin Biotechnol.

